# Gut microbiota–derived metabolite trimethylamine *N*-oxide alters the host epigenome through inhibition of *S*-adenosylhomocysteine hydrolase

**DOI:** 10.1016/j.jbc.2025.110521

**Published:** 2025-07-25

**Authors:** Jessica H. Han, Federico E. Rey, John M. Denu

**Affiliations:** 1 Wisconsin Institute for Discovery, Madison, Wisconsin, USA; 2 Department of Biomolecular Chemistry, University of Wisconsin-Madison, Madison, Wisconsin, USA; 3 Department of Bacteriology, University of Wisconsin-Madison, Madison, Wisconsin, USA; 4 Department of Medical Microbiology and Immunology, University of Wisconsin-Madison, Madison, Wisconsin, USA

**Keywords:** gut microbiome, trimethylamine *N*-oxide, epigenetics, posttranslational modification, one-carbon metabolism, *S*-adenosylhomocysteine hydrolase

## Abstract

The gut microbiota profoundly influences host metabolism through the production of bioactive metabolites that modulate cellular pathways. Among these, trimethylamine *N*-oxide (TMAO) has emerged as an enigmatic molecule linking dietary factors to cellular dysfunction in cardiovascular, neurological, and oncologic disorders. Here, we investigate the cellular and systemic impact of TMAO on metabolic pathways and epigenetic landscapes. Using cultured cells and a mouse model that simulates endogenous TMAO production, we demonstrate that TMAO disrupts the methionine cycle and dynamically remodels chromatin states *via* histone posttranslational methylation and acetylation. Compared to liver, brain cortex and hippocampus show greater sensitivity to TMAO levels. Mechanistically, TMAO noncompetitively inhibits *S*-adenosylhomocysteine hydrolase, leading to accumulation of SAH and subsequent reduction in global methylation capacity. *In vitro* overexpression of SAM synthase, methionine adenosyltransferase 2A, rescues many of these epigenetic defects by boosting SAM/SAH, highlighting the tissue/cell-specific importance of balancing SAM synthesis and SAH clearance. These mechanistic findings reveal that TMAO targets *S*-adenosylhomocysteine hydrolase and disrupts the methionine cycle, expanding our understanding of how gut-derived metabolites modulate chromatin states and identifying potential avenues to mitigate TMAO-associated disease.

The intricate interplay between the gut microbiota and host health has gained substantial attention in recent decades (1, 2, 3, 4). Microbial communities in the large intestine metabolize residual dietary substrates to produce a spectrum of compounds that profoundly affect host physiology and metabolism (2, 5). Factors such as genetics (6), sex (7), diet (5), and environmental influences (8, 9) shape the gut microbiome’s composition, with diet exerting a particularly strong impact (10, 11). Plant-rich diets stimulate the generation of beneficial metabolites, such as short-chain fatty acids (SCFAs) (12, 13, 14), vitamins (15, 16), and phenolic acids (17, 18), whereas diets high in fat (19, 20), sugar (21, 22), and animal proteins (23, 24) can disrupt microbial balance and produce deleterious compounds like methylamines (25, 26, 27) and imidazole propionate (28, 29, 30). These harmful metabolites have been linked to metabolic dysfunction (31, 32, 33, 34), nonalcoholic fatty liver disease (35, 36, 37), and neuroinflammation (38, 39, 40).

Among these microbial products, trimethylamine *N*-oxide (TMAO) has attracted considerable interest for its associations with cardiometabolic (41, 42, 43), neurological diseases (44, 45, 46), and cancer (47, 48, 49, 50). TMAO is formed when gut bacteria metabolize dietary choline and carnitine, followed by hepatic oxidation of trimethylamine (TMA) to TMAO (51, 52). Although elevated TMAO levels consistently correlate with adverse outcomes, the precise mechanisms underlying these associations are not fully understood. Recent studies indicate that TMAO may modulate pathways including G protein–coupled receptor signaling (53, 54) and the protein kinase R-like endoplasmic reticulum kinase (PERK) stress response (27, 55, 56), which in turn impact metabolism and inflammation. However, the influence of TMAO on epigenetic regulation remains underexplored.

Epigenetic states are governed largely by posttranslational modifications (PTMs) of histones, which together with DNA, form the fundamental unit of chromatin, known as the nucleosomes (57, 58, 59). Histones H2A, H2B, H3, and H4 assemble into an octamer, around which approximately 147 bp of DNA wrap, creating a dynamic complex that can be further compacted or relaxed to control gene accessibility (60, 61). The majority of PTMs, including methylation (me), acetylation (ac), phosphorylation, ubiquitination, butyrylation (bu), and propionylation (pr), occur on histone N-terminal “tails” that protrude from this core structure. Depending on which residues are modified, histone PTMs can modulate protein:protein interactions, DNA accessibility, and ultimately gene expression. Methylation on histone lysine residues (K in amino acid nomenclature) can correlate with transcriptional repression or activation depending on the specific site and level of methylation, whereas acetylation typically promotes an open chromatin state conducive to transcription. Because the addition and removal of these modifications require cosubstrates that are central metabolites, changes in acetylation and methylation can be influenced by the levels of these metabolic intermediates.

One-carbon metabolism, in particular, tightly couples nutritional status with epigenetic regulation. Restricting methionine intake lowers cellular levels of SAM, a universal methyl donor, and reduces global histone methylation (62, 63, 64, 65). Conversely, SCFAs such as butyrate and propionate can act as acyl-donors for histone acylation, altering chromatin conformation and transcription (66, 67, 68). Consequently, diet and gut microbial metabolites can reshape epigenetic landscapes by altering cofactor availability for chromatin-modifying enzymes.

Recent research has begun to link TMAO to broader metabolic and epigenetic changes. It has been shown that TMAO activates the NLRP3 inflammasome, possibly promoting tumor growth or metastasis *via* increased vascular endothelial growth factor A, leading to carcinogenesis (47, 49). Moreover, gut-derived TMA production has been linked to changes in DNA methylation, suggesting that TMAO could alter broader epigenetic mechanisms. In gnotobiotic mouse models, high-TMA–producing bacteria induced greater TMAO levels, leading to reduced global DNA methylation and dysregulated one-carbon metabolism (69, 70, 71). Such metabolic imbalances correlated with increased susceptibility to obesity, metabolic disorders, and anxiety (69, 70). These observations suggest an unrecognized target for TMAO in modulating both host metabolism and epigenetic regulation.

Here, we investigate how TMAO interferes with cellular metabolism and elicits epigenetic alterations implicated in disease. We explored the relationship between TMAO and host chromatin dynamics using a mouse model that mimics natural TMAO production, as well as cell cultures exposed to physiologically relevant TMAO concentrations. Chronically high TMAO leads to tissue-specific changes to the proteome and to global changes in chromatin modifications. In a cultured cell model, TMAO induces rapid changes to histone PTMs, involving methylation and acetylation changes. We show that TMAO disrupts the methionine cycle by inhibiting S-adenosylhomocysteine hydrolase (AHCY), leading to SAH accumulation and global reduction in methylation on multiple histone lysine residues. Overexpression (OE) of methionine adenosyltransferase 2A (MAT2A) rescued many of the chromatin changes by restoring the TMAO-induced imbalance in the SAM/SAH ratio. In addition to reduced methylation at specific lysines, increased global histone acetylation at activation-associated sites was a prominent feature in TMAO treatment, which was also mitigated under MAT2A OE. Our results reveal a previously unrecognized mechanism by which the diet-derived microbial metabolite TMAO modulates the epigenome *via* inhibition of an essential enzyme in one-carbon metabolism. These findings offer new insights into the complex interactions between diet, gut microbiome metabolism, and epigenetic modifications, highlighting potential therapeutic targets for TMAO-related diseases.

## Results

### Dietary modulation increases systemic TMAO levels and induces proteomic changes in tissues

**Figure 1:**
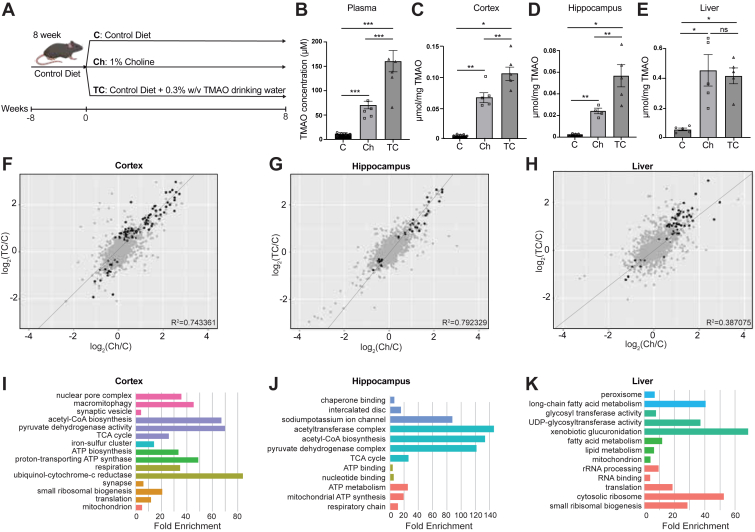
**Dietary modulation increases systemic TMAO levels and induces proteomics changes in tissues.***A*, experimental design using female C57BL/6J mice (n = 5 per group) assigned to three dietary groups: (1) standard chow (control), (2) high-choline diet (1% choline supplementation), and (3) standard chow plus 0.3% w/v TMAO in drinking water. *B*, plasma TMAO concentrations across the three groups. *C–E*, relative TMAO levels in the cortex, hippocampus, and liver. Data are presented with statistical significance indicated as ∗*p* < 0.05, ∗∗*p* < 0.01 (Student’s *t* test). *F*–*H*, correlation plots comparing protein expression between the two high-TMAO groups relative to the control group across the cortex, hippocampus, and liver. Statistically significant proteins (*p* value < 0.05) are highlighted in *black*. TC denotes standard chow plus TMAO in drinking water, Ch denotes the high-choline diet, and C denotes the control diet. *I*–*K*, pathway enrichment analysis of proteins significantly altered in the cortex, hippocampus, and liver, emphasizing biological processes associated with elevated TMAO levels in each tissue. TMAO, trimethylamine N-oxide.

TMAO is a gut microbiota–derived metabolite implicated in metabolic, cardiovascular, and neurological disorders (72, 73, 74, 75, 76, 77, 78, 79), yet its tissue-specific effects are not fully understood. We reasoned that the biological effects of TMAO would be reflected in global changes to proteomic profiles. To investigate alterations to the proteome, elevated TMAO levels in 8-week-old female mice were produced by two different mechanisms: by increasing dietary choline intake, a known TMA precursor and by providing TMAO directly. Mice were conventionally raised and fed with different diets for 8 weeks: (1) standard chow (control), (2) high-choline diet (1% choline supplementation), and (3) standard chow plus 0.3% w/v TMAO in drinking water (Fig. 1*A*). Both the high-choline and TMAO-water groups exhibited significantly higher circulating TMAO levels than controls (Fig. 1*B*). Notably, compared to the high-choline diet, TMAO levels in the TMAO-water group were higher in plasma as well as in the brain cortex and hippocampus (Fig. 1, *B*–*D*). Liver TMAO concentrations were comparable in both experimental groups but significantly higher than the control group (Fig. 1*E*). These results established that dietary choline supplementation and exogenous TMAO intake both elevate systemic TMAO, with particularly pronounced accumulation in the brain under direct TMAO supplementation.

To understand how elevated TMAO levels affect biological pathways in various tissues, we performed label-free quantitative shotgun proteomic analyses on the cortex, hippocampus, and liver from each dietary group. Protein extracts from each tissue were digested and analyzed by LC-MS/MS, generating comprehensive proteomic profiles. We identified a large subset of proteins for which expression significantly differed among the diets (*p* < 0.05). To assess how the two dietary interventions of increasing TMAO levels agreed at the whole proteome scale, correlation plots were generated by comparing the fold change relative to control, with R^2^ values indicating the agreement in diet-specific expression patterns within each tissue type (Fig. 1, *F*–*H*). In the cortex and hippocampus there was a strong correlation (R^2^ = 0.74–0.79), suggesting that TMAO is the principal driver of the biological effects and that TMAO acquired from drinking water or endogenously produced TMAO were largely equivalent in those tissues. In contrast, the much poorer correlation in liver (R^2^ = 0.39) suggests that the mode of TMAO production yields more distinct proteomic signatures. Pathway analysis of the cortex proteome revealed significant enrichment for proteins involved in synaptic transmission, signal transduction, and energy metabolism (Fig. 1*I*) in line with prior work suggesting that TMAO might affect neuronal signaling pathways (76, 80, 81, 82). Several key presynaptic and postsynaptic proteins were highly represented, suggesting possible effects on neurotransmitter release and synaptic plasticity.

In the hippocampus, proteins associated with learning and memory processes, oxidative stress response, and mitochondrial function were significantly affected (Fig. 1*J*), supporting recent work that connects TMAO to neuroinflammation and synaptic dysfunction (75, 76, 82, 83, 84, 85). Enrichment of proteins involved in synaptic efficacy and long-term potentiation such as postsynaptic density proteins and Ca^2+^-calmodulin–dependent kinases points to potential modifications of hippocampal plasticity under high TMAO conditions. Enzymes related to reactive oxygen species detoxification were also enriched, suggesting oxidative stress modulation in the hippocampus.

In the liver, proteomic alterations highlighted enzymes involved in peroxisome function and glycosyl transferase activity, both crucial for xenobiotic detoxification (Fig. 1*K*). Several differentially expressed proteins also participate in fatty acid and lipid metabolism, while changes in mitochondrial and oxidative phosphorylation components further pointed to a potential impact on hepatic energy homeostasis and redox balance (86, 87, 88). Collectively, these findings indicate that both dietary choline supplementation and exogenous TMAO administration elevate systemic TMAO concentrations and drive tissue-specific proteomic remodeling, implying that TMAO’s potential role in dietary-microbial link that reshapes biological pathways.

### High TMAO elicits tissue-specific histone PTM patterns and unique metabolite profiles

**Figure 2:**
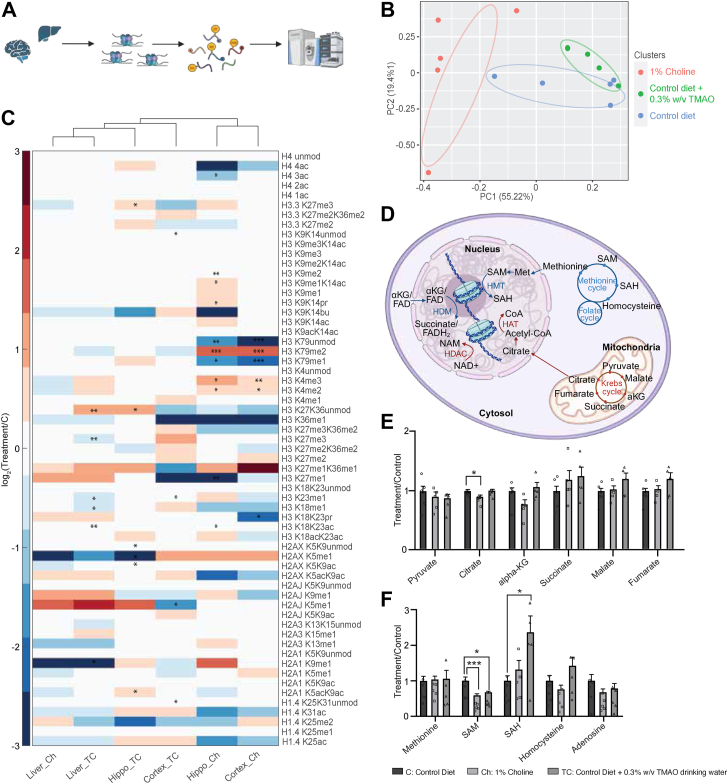
**High TMAO elicits tissue-specific histone PTM patterns and unique metabolite profiles.***A*, schematic illustrating the experimental workflow for histone extraction, chemical derivatization, and posttranslational modification (PTM) profiling using tissues harvested from mice. *B*, principal component analysis of histone PTM profiles in hippocampus. Each point represents an individual sample. Ellipses denote the 95% confidence intervals for each dietary treatment group, illustrating the spread and clustering of the data. Colors correspond to the three different dietary interventions. *C*, hierarchical clustering heatmaps of log2 fold change in stoichiometric histone peptide proteoforms for the cortex, hippocampus, and liver, respectively, relative to mice fed on a control diet. Data were obtained *via* LC-MS/MS (n ≥ 4). Statistical significance is indicated as ∗*p* < 0.05, ∗∗*p* < 0.01, and ∗∗∗*p* < 0.005 (Welch’s *t* test). *D*, illustration of key metabolic pathways that furnish substrates and cofactors for epigenetic reactions inside the cells. *E*-*F*, bar plots showing relative levels of select Krebs cycle intermediates and methionine cycle metabolites, respectively, involved in methylation and acetylation processes. Data are presented with statistical significance indicated as ∗*p* < 0.05, ∗∗*p* < 0.01, and ∗∗∗*p* < 0.005 (Student’s *t* test). PTM, posttranslational modification; TMAO, trimethylamine N-oxide.

To determine whether TMAO-induced proteomic shifts are linked to global changes in epigenetic state of tissues, we profiled histone PTMs in the cortex, hippocampus, and liver. Following tissue harvest, samples were prepared by trypsinization, labeled with heavy acetic anhydride and histone PTMs were quantified using on tandem mass spectrometry (Fig. 2*A*). Overall, elevated TMAO levels were associated with pronounced alterations in the histone PTM landscape, with the hippocampus and cortex displaying more dramatic changes compared to the liver. Principal component analysis of histone PTM profiles revealed distinct clustering by tissue and diet, indicating both inherent epigenetic differences across the three tissues and diet-specific modulations of chromatin states (Figs. 2*B* and S1*A*). Within each tissue, high-choline and TMAO-supplemented groups formed discrete sub-clusters, suggesting diet- and tissue-dependent regulation of histone modifications.

A hierarchical heatmap of histone PTMs revealed that, in the liver, the two experimental groups (high-choline diet (Ch) and TMAO-water (TC)) shared broadly similar modification profiles (Fig. 2*C*). Certain changes were consistent in both, such as decreased levels of H2A1 K9me1, H2A3 K13me1, H2AX K5me1, H3 K36me1, and H3 K9K14bu, and increased levels of H2AJ K5me1, H2AX K5acK9ac, H3 K27me1, and H3 K27me1K36me1. The TMAO-water group (TC) showed more significantly altered sites overall, particularly on histone H3, including increased H3 K18K23ac and canonical H3 K27K36 but decreased H3 K18me1, H3 K23me1, and H3 K27me3. Interestingly, the altered epigenomes in hippocampus and cortex within the TC groups clustered closely to each other and to the liver groups. Notable hippocampal changes in the TC group included significantly increased H2A1 K5acK9ac, H2AX K5K9ac, and H2AX K5K9, coupled with decreased H2AX K5me1. In the cortex, H1.4 K25K31 and H3 K9K14 were upregulated, while H2AJ K5me1, H3 K23me1, H3 K27me1, and H3 K36me1 decreased markedly. The high-choline group (Ch) also demonstrated distinct epigenetic patterns in the hippocampus and cortex. H3 K18K23ac, H3 K4me2/me3, H3 K79me2, and H3 K9K14bu, and H3 K9me1K14ac were significantly elevated, whereas H3 K27me1, H3 K36me1, H3 K79me1, and H4 4ac were reduced. Collectively, these changes suggest that although the liver shares some epigenetic shifts between high-choline and TMAO-water groups, each tissue exhibits unique histone PTM signatures in response to elevated TMAO or choline.

Building on these epigenetic data, we next examined whether TMAO influences systemic metabolic pathways that intersect with chromatin regulation. We quantified Krebs cycle and methionine cycle metabolites in plasma using LC-MS (Fig. 2*D*). These metabolites serve as intermediates for direct substrates and/or inhibitors in numerous chromatin-modifying enzyme reactions (89, 90, 91). For instance, alpha-ketoglutarate functions as a required cosubstrate for a subset of Jumonji domain histone demethylases (92) and ten-eleven translocation DNA demethylases (93), whereas succinate and fumarate can competitively inhibit these α-ketoglutarate–dependent enzymes and stabilize repressive methyl marks (94, 95). Elevated concentrations of acetyl-CoA, an important intermediate derived from the Krebs cycle, are also linked to an increase in histone acetylation levels (96, 97). Additionally, methionine and ATP are used to generate SAM, the universal methyl donor in cellular methylation reactions (62, 98). The product of SAM-dependent methylation, SAH, can act as a potent inhibitor of methyltransferases (99, 100, 101). In a major process to recycle methionine, SAH is subsequently hydrolyzed to adenosine and homocysteine (102, 103).

Within the Krebs cycle, only citrate was significantly altered in high-choline–fed mice (Fig. 2*E*), with a decrease of 10% compared to control. Within the methionine cycle, SAM levels were reduced by approximately 50%, while the methylation product SAH was significantly elevated by 2-fold in the TC group compared to the control group (Fig. 2*F*). These shifts suggest that elevated TMAO conditions may disrupt one-carbon metabolism, particularly the methionine cycle, potentially diminishing methyl-group availability for chromatin modifications. These metabolic and epigenetic findings indicate that TMAO-driven perturbations may reshape the epigenome by altering key cofactor and substrate pools that underlie histone and DNA methylation.

### TMAO treatment leads to altered methionine cycle and epigenetic states in cells

To explore how TMAO affects the methionine cycle and chromatin regulation at the molecular level, we turned our investigations toward well-established cell line models and treated several human cell lines—MCF7 (breast cancer), HCT116 (colorectal cancer), and HEK293T (kidney)—with various concentrations of TMAO. Cells were acclimated in fresh media for 1 h to minimize potential confounders and then refreshed with either TMAO-containing or vehicle-only media (Fig. S2*A*). Cell viability and proliferation was assessed and showed no significant reduction in viable cell numbers across the tested TMAO range from 1 μM to 50 mM over 24 h (Fig. S2, *B* and *C*). These results confirm that TMAO is not cytotoxic under these experimental conditions, supporting the use of common cultured cells as suitable for exploring downstream metabolic and epigenetic effects.

**Figure 3:**
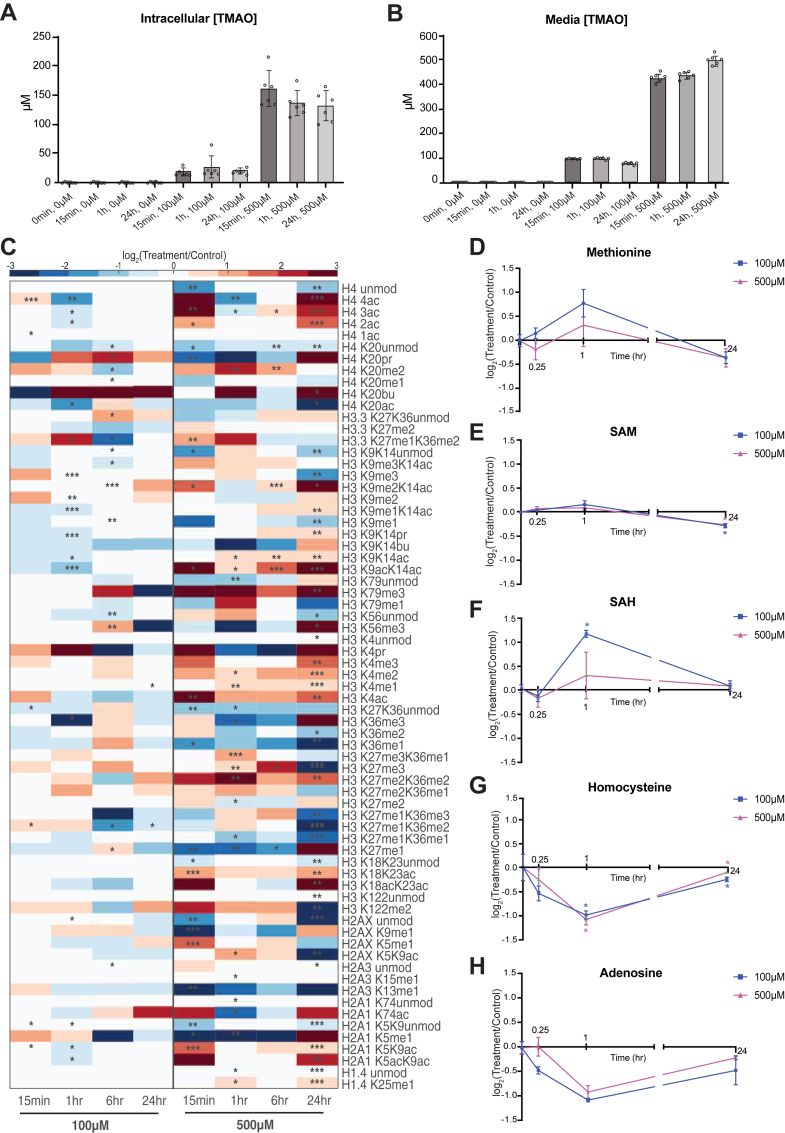
**TMAO treatment leads to altered methionine cycle and epigenetic states in cells.***A*, time-course measurements of TMAO uptake by HCT116 cells at different doses. *B*, TMAO concentrations in the culture media of HCT116 cells. *C*, heat maps of log_2_ fold change in stoichiometric histone peptide proteoforms in HCT116 cells treated with 500 μM TMAO relative to vehicle controls (n ≥ 4). Statistical significance is indicated as ∗*p* < 0.05, ∗∗*p* < 0.01, and ∗∗∗*p* < 0.005 (Welch’s *t* test). *D*–*H*, time-series analysis of key methionine cycle metabolites in HCT116 cells exposed to 100 μM or 500 μM TMAO relative to vehicle control. ∗*p* < 0.05 (Student’s *t* test). TMAO, trimethylamine *N*-oxide.

Next, we measured the capacity of cells to take up TMAO as a function of time at various concentrations. TMAO was detectable in cells as early as 15 min posttreatment in a dose-dependent manner, whereas untreated controls showed no measurable TMAO (Figs. 3*A* and S2, *D*–*F*). TMAO levels in the culture media remained stable, indicating that, once equilibrated, TMAO is not appreciably metabolized or degraded in the media (Fig. 3*B*). The lone exception was HepG2 cells which appeared to show approximately 50% reduced levels after 1 h (Fig. S2*E*). Because HCT116 cells showed both rapid TMAO uptake and steady intracellular levels, we chose this cell line for subsequent mechanistic experiments.

To determine whether TMAO induces global changes to chromatin states in a cell culture model, HCT116 cells were treated with 100 μM or 500 μM TMAO for up to 24 h and histone PTMs were profiled as in the prior mouse tissue analysis (Fig. 3*C*). Both concentrations of TMAO led to significant changes to histone PTMs as early as 15 min. At 100 μM TMAO, the overall chromatin changes were less robust than the 500 μM treatment, showing more significant alterations between 1 and 6 h before partially reverting by 24 h. In the 500 μM treatment, TMAO elicited more pronounced and sustained shifts in methylation and acetylation. Several methylation marks, including H2A3 K15me1, H3 K27me1, H3 K27me1K36me1/2, H3 K36me3, and H3 K9me1 showed a clear trend toward reduction. Also, several acetylation marks, including diacetylated, triacetylated, and tetra-acetylated H4, H3K9acK14ac, and H2AK5K9ac were elevated, pointing to a shift in the balance of opposing chromatin-modifying enzymes.

To assess whether TMAO induces metabolic changes consistent with the *in vivo* observations, HCT116 cells were treated with 100 μM or 500 μM TMAO for up to 24 h and LC-MS quantification of SAM-related metabolites was performed (Fig. 3, *D*–*H*). Similar to the results in mice, TMAO treatment of cells caused perturbations in the methionine cycle. At 1 h, SAH levels increased by 2-fold, while homocysteine and adenosine both decreased to a similar degree compared with vehicle-treated controls at the same time point (Fig. 3, *F*–*H*). Methionine levels were not significantly changed, although levels tended to increase at 1 h and were slightly reduced at 24 h. SAM levels were significantly reduced by 24 h. Homocysteine and adenosine concentrations rebounded over time toward the control, though both were slightly lower than control at 24 h. Altogether, these data suggest that TMAO disrupts portions of methylation processes by transiently increasing SAH and reducing homocysteine and adenosine.

TMAO has been reported to bind and activate PERK, a mediator of the unfolded protein response that phosphorylates eukaryotic translation initiation factor 2α (eIF2α) (27, 104, 105). This phosphorylation generally lowers global protein synthesis during endoplasmic reticulum stress (55). To assess whether the TMAO-induced chromatin remodeling arose from the translational suppression of chromatin-modifying enzymes mediated by PERK-eIF2α signaling, we monitored phosphorylated eIF2α by Western blot (Fig. S2, *G*–*I*). Although HCT116 cells exposed to 100 μM TMAO displayed significant changes in histone PTMs within the first hour, and those treated with 500 μM showed significant changes at 15 min (Fig. 3*C*), there was no evidence of PERK-eIF2α signaling during this period (Fig. S2*H*). We did note a small increase in eIF2α phosphorylation in the 24-h time point at 100 μM; however, this was the exception and the data taken together suggest that the rapid chromatin alterations do not involve the PERK–eIF2α pathway. Instead, TMAO appears to provoke early epigenetic modifications *via* a distinct mechanism that perturbs the methionine cycle and induces dose- and time-dependent shifts in histone PTMs.

### TMAO inhibits AHCY activity

**Figure 4:**
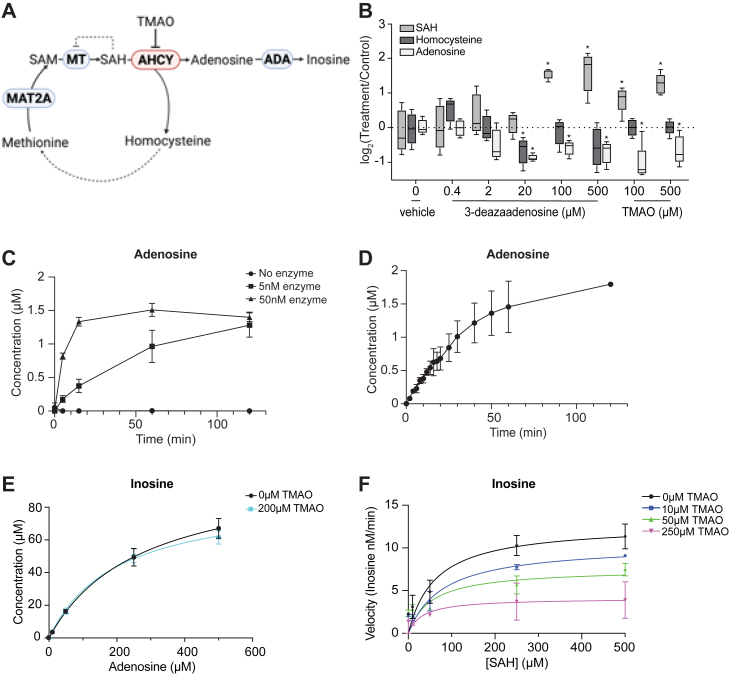
**TMAO inhibits SAH hydrolase activity.***A*, hypothesis model illustrating TMAO inhibition of S-adenosylhomocysteine hydrolase (AHCY) in the methionine cycle. *B*, comparative analysis of SAH, homocysteine, and adenosine in HCT116 cells treated with various concentrations of 3-deazaadenosine (3DA) or TMAO for 30 min. Data are presented with statistical significance indicated as ∗*p* < 0.05 (Student’s *t* test). *C*, quantification of adenosine to assess AHCY activity at three enzyme concentrations using 50 μM SAH as the substrate over a 2-h span. *D*, time course of adenosine formation using 5 nM AHCY to establish a linear reaction phase steady state. *E*, quantification of inosine to assess the effects of TMAO in adenosine deaminase that irreversibly converts adenosine to inosine. *F*, quantification of inosine in the presence of AHCY, ADA, and increasing concentrations of TMAO. TMAO, trimethylamine *N*-oxide.

The fact that SAM and methionine metabolic pathways were consistently altered in both mice and cell culture experiments under elevated TMAO, and that histone PTM patterns, particularly methylation, were dramatically affected, led us to postulate that TMAO might directly or indirectly affect enzymes in these pathways. Specifically, we focused our attention on AHCY given that AHCY is the sole mammalian enzyme that converts SAH into homocysteine and adenosine (106, 107, 108, 109) (Fig. 4*A*). The most consistent trends in the metabolomics data were the buildup of SAH and the reduction of homocysteine and adenosine. These observations led us to propose that TMAO acts as an inhibitor of AHCY. Partial inhibition of AHCY could lead to SAH buildup, which in turn serves as a potent product inhibitor of numerous methyltransferases (Fig. 4*A*). To determine whether TMAO’s metabolic effects mirror those of an established AHCY inhibitor, we treated cells with increasing concentrations of 3-deazaadenosine (3DA) and compared the metabolite changes to those observed with TMAO treatment. Within 30 min, TMAO induced changes in homocysteine, adenosine, and SAH levels that closely matched the trends seen under 3DA treatment (Figs. 4*B* and S3*A*). Specifically, adenosine and homocysteine decreased significantly at ≥20 μM 3DA, while SAH levels increased. These parallels suggest that TMAO, like 3DA, exerts its cellular effects *via* AHCY inhibition. When comparing the corresponding histone PTM profiles from cells exposed to 50 μM 3DA for 30 min and those exposed to 500 μM TMAO for 15 min, we found a positive though modest correlation (Fig. S3*B*). Together, these data supported a hypothesis that TMAO directly inhibits AHCY.

To substantiate TMAO’s inhibitory role in AHCY, we cloned, expressed, and purified recombinant human AHCY (Fig. S3*C*) and investigated whether TMAO alters AHCY catalytic activity. Initial characterization of purified AHCY revealed that SAH is converted to adenosine in an enzyme dependent fashion, with rates of product formation dependent of levels of AHCY. Increased enzyme concentration accelerated adenosine production, whereas no significant adenosine formed in the absence of AHCY (Fig. 4*C*). We then established the linear reaction phase (steady state) is within 12 min, during which substrate depletion and product accumulation had negligible effects on the steady-state rates (Fig. 4*D*) and verified that adenosine production did not occur *via* nonenzymatic hydrolysis (Fig. S3*D*). To prevent feedback production inhibition, we included adenosine deaminase (ADA) to irreversibly convert adenosine into inosine (110) (Fig. 4*A*). ADA activity was unaffected by TMAO, and adenosine was fully converted to inosine within minutes (Figs. 4*E* and S3, *E* and *F*). It is important to note that TMAO-treated cells showed no change in AHCY protein levels, implying that TMAO targets AHCY activity rather than protein abundance (Fig. S3*G*). Additionally, adenosine quantification remained unchanged when adenosine was incubated with or without TMAO (Fig. S3*H*), confirming that TMAO does not affect the ability to accurately measure products directly. Under these optimized conditions, we performed a steady-state inhibition experiment where TMAO and SAH substrate concentrations are varied, and the resulting rates were measured and analyzed for potency and mechanism. TMAO caused a dose-dependent decline in AHCY activity (Fig. 4*F*, Table), and fitting the kinetic data to various models of inhibition revealed that TMAO acts as a noncompetitive inhibition with a K_i_ of 36.5 μM 95% confidence interval [21.4, 65.8]. Together, these data suggest that the effects of TMAO stem from a direct, noncompetitive inhibition of AHCY rather than changes in AHCY expression, ultimately disrupting SAH turnover and perturbing one-carbon metabolism.

### MAT2A OE restores methylation potential and partially restores disrupted histone modifications

**Figure 5:**
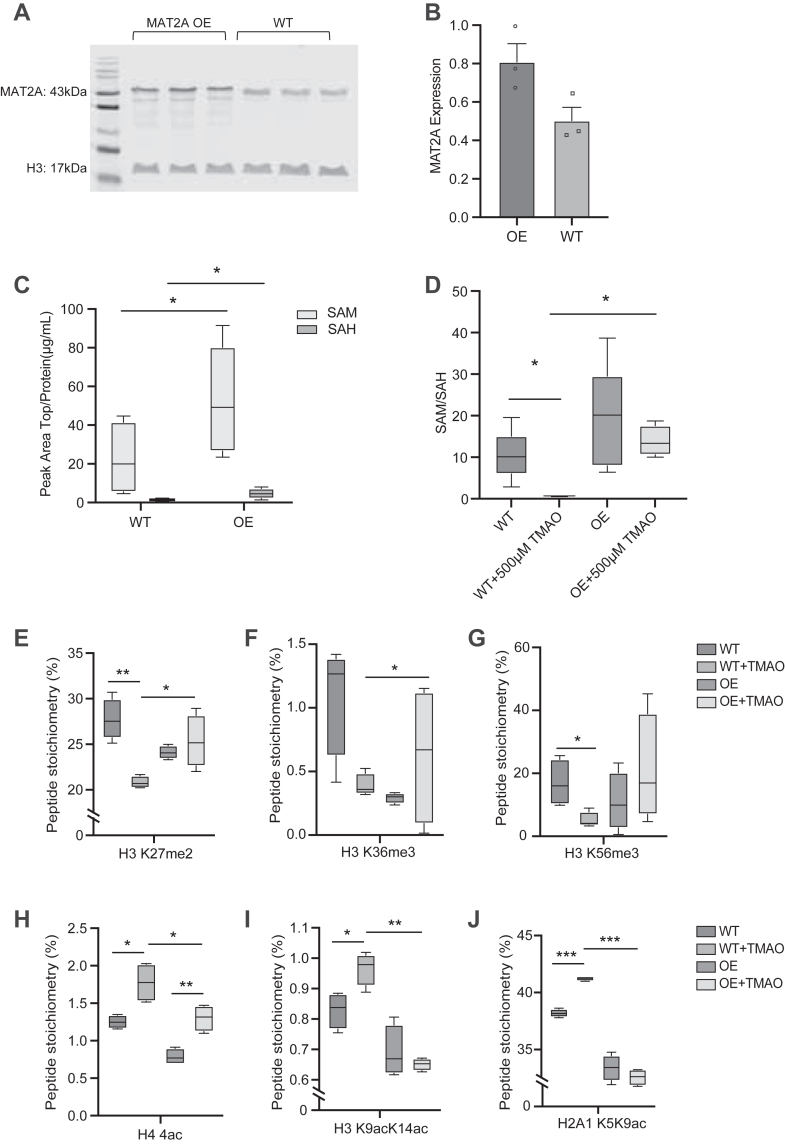
**MAT2A overexpression restores methylation potential and partially rescues disrupted histone methylation.***A* and *B*, Western blot analysis of MAT2A protein level in MAT2A overexpressing cells (OE) and WT cells. *C*, Comparative analysis of SAM and SAH in WT and OE treated with or without 500 μM TMAO for 30 min. Data are presented with statistical significance indicated as ∗*p* < 0.05 (Student’s *t* test). *D*, relative SAM:SAH in WT and OE treated with or without 500 μM TMAO. *E*–*J*, peptide stoichiometry of H3 K27me2, H3 K36me3, H3 K56me3, H4 tetra-ac, H3 K9acK14ac, and H2A1 K5K9ac in WT and OE treated with or without 500 μM TMAO for 30 min, respectively. MAT2A, methionine adenosyltransferase 2A; TMAO, trimethylamine *N*-oxide.

To determine whether increasing SAM production could reverse TMAO-induced epigenetic disruption *via* AHCY inhibition, we overexpressed the MAT2A in HCT116 cells. Western blot analysis confirmed that MAT2A protein levels were approximately doubled in the OE cells compared to WT cells (Fig. 5, *A* and *B*). To link these changes in MAT2A OE to altered methylation capacity, we quantified intracellular SAM and SAH levels in both OE and WT cells. Consistent with earlier observations, TMAO treatment elevated SAH in WT cells (Figs. 3*F* and 4*B*), whereas OE cells maintained higher concentrations of both SAM and SAH than the corresponding WT cells (Fig. 5*C*). This suggests that augmenting MAT2A drives increased flux through the methionine cycle. When we examined the SAM/SAH ratio across treatments, TMAO significantly reduced this ratio in WT cells, implying a loss of methylation potential (Fig. 5*D*). In contrast, the SAM/SAH ratio in OE cells remained relatively stable upon TMAO treatment, indicating that the increased SAM production compensates for the partial AHCY block. Indeed, OE cells treated with TMAO retained a significantly higher SAM/SAH than TMAO-treated WT cells, implying that although TMAO does elevate SAH *via* AHCY inhibition, the increased production of SAM offsets this increase to maintain SAM/SAH.

Next, we asked whether maintaining methylation potential through MAT2A OE could alleviate TMAO-induced epigenetic changes. A histone PTM profiling assay (Fig. S4*A*) revealed that several histone methylation marks diminished in TMAO-treated WT cells were partially or fully restored in OE cells. While some sites remained sensitive to TMAO even with MAT2A OE, a subset of methylation marks returned to near-baseline levels in OE+TMAO compared to WT+TMAO. Notably, MAT2A OE partially restored H3 K27me2, H3 K36me3, H3 K56me3, H3 K64me2, H3.3 K36me1, and H4 K31me2 (Figs. 5, *E*–*G* and S4, *B* and *C*). As observed in the time course (Fig. 3*C*), TMAO-treated cells also exhibited notable shifts in histone acylation. H2A1 K5K9ac, H3 K9acK14ac, and general H4 acetylation were induced by TMAO in WT cells (Fig. 5, *H*–*J*). Similarly, acylation levels declined in OE+TMAO cells relative to WT+TMAO, suggesting that maintaining the SAM/SAH can restore the hyperacetylation caused by TMAO. Overall, these findings reveal an intricate link between methylation and acetylation pathways and demonstrate that elevating SAM through MAT2A OE can partially counteract TMAO’s disruptive effects on chromatin.

## Discussion

Here, we provide a detailed investigation into how dietary and endogenously produced TMAO disrupts one-carbon metabolism and reshapes the epigenetic landscape, likely *via* AHCY inhibition. TMAO significantly elevates circulating levels of SAH *in vivo* and decreases SAM/SAH in cells, reducing overall methyl-donor availability and culminating in widespread changes in histone methylation and acetylation. This study demonstrates a direct link between the gut microbiota–derived metabolite TMAO and the modulation of chromatin states across multiple tissues and cell culture models, revealing a novel mechanism that modulates host metabolism and chromatin state.

The combined proteomic analyses revealed that TMAO exerts distinct outcomes in the cortex, hippocampus, and liver. Compared with liver, brain tissues were remarkably sensitive to TMAO as evidenced by the number of significantly altered histone PTMs and whole proteome changes. There were marked alterations in synaptic and mitochondrial pathways, suggesting that shifts in SAM metabolism influence cognitive or neuroinflammatory processes. These data align with previous reports linking TMAO to neurodegenerative and neuroinflammatory phenotypes under metabolic stress (76, 80, 81, 82). In contrast, the liver displayed a more nuanced response (chromatin and proteome), and interestingly, there was a poorer correlation between the effects of TMAO administered in water *versus* a high-choline diet. This difference in liver might reflect the liver’s natural capacity to convert TMA to TMAO, *via* hepatic flavin monooxygenases activity (111, 112). Although we did not directly assess gut microbiome composition in this study, it is well documented that diet modulates microbial composition and function (11, 113, 114) and elevated dietary choline can enrich TMA-producing bacteria (115, 116). Importantly, our data indicate that choline supplementation was sufficient to increase circulating TMAO levels. In the high-choline group, TMA is microbially generated in the colon and subsequently delivered to the liver *via* the portal circulation, where it is oxidized to TMAO. In contrast, the TMAO-in-water group can be absorbed throughout the gastrointestinal tract, bypassing microbial and hepatic conversion. Despite these differences, we observed comparable TMAO levels in the liver between the two treatment groups, suggesting that hepatic TMAO accumulation may reach a saturation threshold beyond which additional substrate or circulating TMAO does not further increase liver concentrations. Meanwhile, higher TMAO levels in the cortex and hippocampus of the TMAO-in-water group suggest more efficient systemic and brain distribution of TMAO.

The gut–brain axis provides a bidirectional communication network encompassing neural, endocrine, and immune signaling through which microbial metabolites can affect brain function and behavior (113, 114, 115, 116, 117). Here, we demonstrate that the proteomes and chromatin states in cortex and hippocampus are remarkably remodeled under high TMAO, providing new clues to the impact on cognition and neurological health. At this point it is unclear why the brain shows greater sensitivity, but such tissue-specific responses underscore that local enzyme activities (*e.g.,* methyltransferases, methionine cycle enzymes like AHCY), substrate availabilities, and chromatin status collectively are likely to dictate the magnitude and direction of TMAO-driven responses. These considerations may explain why certain histone residues, such as H3 K27me2, H3 K36me3, and H3 K56me3, are especially vulnerable to TMAO in the cell culture model, while other marks remain partially intact. Our data show that TMAO is a noncompetitive inhibitor of AHCY, resulting in a buildup in SAH that has been reported as a feedback inhibitor of methyltransferases (118, 119, 120). Notably, this mechanism parallels what has been observed under other methyl-depleting conditions, where high SAH levels drastically limit histone and DNA methylation (62, 63, 64, 65).

One key observation is that overexpressing the MAT2A partially rescues global chromatin changes induced by TMAO. Despite the ability of TMAO to still inhibit AHCY, MAT2A OE enhances SAM production, offsetting some of the epigenetic disruption by maintaining SAM/SAH. This is possible because methionine is available from the media. Notably, some histone marks remain sensitive to elevated SAH, suggesting different methyltransferases and histone residues can have varied sensitivities to SAM depletion and SAH accumulation. Moreover, certain methylation marks may be more quickly lost (*via* the presence of specific demethylases) or gained in response to metabolic perturbance, while others may require sustained changes to enzyme activity for a noticeable shift. Thus, it is logical that MAT2A OE partially restores a subset of methyl marks but not all. This selective rescue may reflect the specific kinetics, substrate affinities, and distinct regulatory mechanisms of the methyltransferases involved.

In addition to changes in methylation, TMAO treatment induced strong hyperacetylation at several sites that are associated with increased chromatin accessible and gene activation. The mechanism, which appears to be restored under MAT2A OE, is unclear but could be in response to direct changes in chromatin methylation, or as a consequence of a metabolic disruption caused by acyl inhibition. TMAO leads to transient increases in SAH and decreases in homocysteine and adenosine. Homocysteine is also a precursor in the transsulfuration pathway that generates cysteine and glutathione. Adenosine can have several fates but can be phosphorylated to AMP which has been linked to pathways of H3 deacetylation (121, 122). Thus, a TMAO-dependent decrease in adenosine could explain the increased histone acetylation. Acetylation reactions require acyl-CoA donors, which are tied to fatty acid metabolism and overall acetyl-CoA availability (96, 97). Lower circulating citrate levels in TMAO-treated mice might suggest that the citrate is more rapidly converted to acetyl-CoA in the cytoplasm by ATP citrate lyase. Further studies will be needed to uncover the actual mechanisms by which TMAO induces hyperacetylation.

Our findings tie into broader questions about how cells adapt chromatin states under metabolic stress. We show here that some epigenetic marks endure in TMAO-rich environments, especially when SAM synthesis is increased. This capacity for selective chromatin maintenance may be essential for preserving genomic integrity and preventing the derepression of transposable elements (123, 124, 125). In chronic diseases or advanced age, loss of these compensatory mechanisms could compound the adverse effects of elevated TMAO, as evidenced by its associations with atherosclerosis (42, 51, 104, 126) and other metabolic disorders (20, 27, 31).

Several key questions arise from this work. First, additional studies are needed to clarify how TMAO’s inhibition of AHCY intersects with other choline-derived metabolites such as betaine and phosphatidylcholine that also influence the methionine cycle. Second, determining whether TMAO’s epigenetic disruption persists over long periods and is reversible will be pivotal for understanding its role in chronic diseases. Finally, strategies to mitigate TMAO-related harm, such as pharmacological inhibition of TMAO production or targeted enhancement of SAM biosynthesis, could serve as promising interventions in metabolically stressed or aged populations. Future mechanistic studies focusing on specific histone marks would be especially instructive. For instance, performing high-resolution ChIP-seq on residues such as H3 K27me2, H3 K36me3, H3 K9ac, and H3 K14ac in *AHCY*-KO or TMAO-treated mice may help pinpoint the genomic regions most susceptible to AHCY inhibition. Parallel incorporation of ^13^C-methionine could further elucidate real-time methyl-group flux under TMAO-mediated or *AHCY* KO-induced stress. Moreover, exploring whether TMA-lyase inhibitor can reduce TMAO production in *AHCY*-knockout systems and thus ameliorate TMAO’s epigenetic effects would be a valuable step toward translation applications.

In summary, the coordinated alteration of chromatin modifications under TMAO-induced metabolic stress highlights the dynamic interplay between diet-derived microbial metabolites, epigenetic enzymes, and chromatin states. By delineating the mechanism whereby TMAO inhibits AHCY and disrupts one-carbon metabolism, our study emphasizes the importance of proper control of SAM-associated metabolites to maintain methylation fidelity and epigenetic integrity.

## Experimental procedures

### TMAO treatment in mice and cultured cells

#### Mice and diets

The experimental procedures conducted on all mice were conformed with the animal use protocols approved by the University of Wisconsin–Madison Animal Care and Use Committee. Female C57BL/6J mice, aged 7 weeks, were procured from The Jackson Laboratory. These mice were then bred under specific pathogen-free conditions at the University of Wisconsin–Madison Biomedical Research Models Services facility.

The mice were housed on a 12-h light/dark cycle and had unrestricted access to rodent chow for an initial week. At the onset of their eighth week, they were divided into three dietary groups: a chow diet group (n = 5), a high-choline diet group (1%, n = 5), and a group on a chow diet supplemented with TMAO in drinking water (0.3% w/v, n = 5). The mice were given unrestricted access to the TMAO for approximately 8 weeks. Following this period, mice were euthanized for sample collection.

Mice underwent cardiac puncture for terminal blood collection under isoflurane anesthesia, followed by cervical dislocation. Subsequent to removal, the brain was swiftly submerged in ice-cold PBS. Dissection of brain sections into the cortex and hippocampus was executed, and various tissues were immediately frozen in liquid nitrogen. Concurrently, blood samples were centrifuged at 3000×*g* for 15 min at 4 °C, after which the plasma layer was extracted and snap-frozen. All snap-frozen samples were preserved at −80 °C until further analysis. It should be noted that all tissue collection procedures were carried out between the hours of 7 AM and 10 AM.

### TMAO cell line treatments

HCT116 (American Type Culture Collection [ATCC], #CCL-247), HepG2 (ATCC, #HB-9086), MCF7 (ATCC, #HTB-22), and HEK293T (ATCC, #CRL-1573) cell lines were cultured under 37 °C and 5.0% CO2. For TMAO treatments, cells were acclimated in fresh, growing media 1 h prior to TMAO exposure. TMAO solutions were freshly prepared in deionized water on the day of the experiment.

### Metabolite extraction and quantification

#### Metabolite extraction from plasma

Plasma TMAO concentrations were determined using LC-MS/MS. An amalgamation of one volume of plasma and four volumes of ice-cold extraction solution, containing 2.5 μM d9-TMAO internal control in HPLC-grade methanol, was subjected to centrifugation at 21,000×*g* for 3 min at 4 °C.

For TMAO level evaluation in cultured cells, the cells were cultivated in the growing media. TMAO solutions were freshly prepared in deionized water on the experiment day. Post-TMAO incubation, cells were washed twice with PBS and metabolites were extracted using 1400 μl of the aforementioned ice-cold extraction 80% methanol. Following this, the resultant solution was centrifuged at 21,000×*g* for 5 min at 4 °C.

For further processing, 600 μM of the supernatant from each sample was transferred to two sets of 1.5 ml Eppendorf microcentrifuge tubes and thoroughly dried using a Thermo Fisher Scientific Savant ISS110 SpeedVac. The dried metabolite extracts were stored at −80 °C.

### Metabolite extraction from tissues

For *Mus musculus* liver tissue, an average weight of 30 mg of tissue was transferred into an individual 1.5 ml Eppendorf microcentrifuge tube and dounce-homogenized in 200 μl of −20 °C 80:20 MeOH:H_2_O extraction mixture on ice. The tissue homogenate was subjected to a 5-min ice incubation following vortexing and then centrifuged at 21,000×*g* for 5 min at 4 °C. The supernatant was relocated to a separate Eppendorf tube. The remaining pellet was then resuspended in 100 μl of −20 °C 40:40:20 ACN:MeOH:H_2_O extraction solvent, followed by a 5-min ice incubation. The tissue homogenate was centrifuged again at 21,000×*g* for 5 min at 4 °C, and the resulting supernatant was combined with the previously separated metabolite fraction. The unified metabolite extract for each sample was transferred to a 1.5 ml Eppendorf microcentrifuge tube and thoroughly dried using a Thermo Fisher Scientific Savant ISS110 SpeedVac. The dried metabolite extracts were stored at −80 °C.

### Metabolite extraction from cells

For cell lines, around 1.5 × 10^6^ cells were swiftly rinsed twice with 2 ml of ice-cold PBS at pH 7.4, prior to adding 1.5 ml of −80 °C 80:20 MeOH:H_2_O extraction solvent. Cells were incubated with the extraction solvent at −80 °C for 15 min, then scraped off their dish, and transferred into a 2 ml Eppendorf microcentrifuge tube. This was followed by centrifugation at 21,000×*g* for 5 min at 4 °C. The supernatant was transferred to a new 2 ml Eppendorf microcentrifuge tube. The supernatant was completely dried using a Thermo Fisher Scientific Savant ISS110 SpeedVac. The dried metabolite extracts were stored at −80 °C.

### TMAO quantification

The dried metabolite extracts were resuspended in one volume of liquid chromatography (LC)-graded water and microcentrifuged for 5 min at 21,000×*g* at 4 °C to sediment any insoluble residue. The supernatant was thereafter transferred to a glass vial for LC-MS analysis.

The samples were loaded onto a Waters ACQUITY C18 ultra-performance liquid chromatography (UPLC) column (1.7 μm, 2.1 mm × 100 mm) that was linked to a Thermo Fisher Scientific Q-Exactive mass spectrometer, operated at a flow rate of 0.2 ml/min. Samples eluted over a 7-min isocratic gradient of 25% water + 5 mM ammonium acetate + 0.05% acetic acid and 75% methanol. TMAO was quantified in the positive mode employing parallel reaction monitoring using an inclusion list of 76.076 and 85.132 *m/z* for TMAO and d9-TMAO, respectively. Peak quantification was executed with El-Maven, and internal standards were employed for plasma concentration calculations.

### LC-MS metabolite analysis

For unbiased metabolite analyses, the dried metabolite extracts were resuspended in one volume of LC-graded water and centrifuged for 5 min at 21,000×*g* at 4 °C to sediment any insoluble residue. The supernatant was thereafter transferred to a glass vial for LC-MS analysis.

The samples were injected onto a Dionex Ultimate3000 nanoflow HPLC, which was equipped with a Waters ACQUITY UPLC BEH C18 column (2.1 mm × 100 mm) and was connected to a Thermo Fisher Scientific Q-Exactive mass spectrometer, operating at a flow rate of 0.2 ml/min. In the case of samples processed in negative mode, the mobile phase was composed of methanol (A) and water supplemented with 3% methanol, 10 mM tributylamine, and 0.05% acetic acid (B). A linear gradient from 95% to 5% B was implemented over 15 min to resolve the metabolites. Only primary ion from mass spectrometry (MS1) data was captured with a scan resolution of 70,000, automatic gain control target set to 1 × 10^6^, and a scan range spanning from 59 to 880 *m/z*.

For methionine cycle metabolites, each prepared dried metabolite extracts, diluted in 85% acetonitrile (ACN), and was injected into a Thermo Fisher Scientific Vanquish UHPLC paired with a Waters XBridge BEH Amide column (100 mm × 2.1 mm, 3.5 μm) and a Thermo Fisher Scientific Q-Exactive mass spectrometer. The aqueous phase (A) consisted of 97% H_2_O, 3% ACN, 20 mM ammonium acetate, and 15 mM ammonium hydroxide with a pH of 9.6, while the organic phase (B) was 100% ACN. The metabolites were resolved by applying a linear gradient as follows: 85% B at 0.15 ml/min for 1.5 min; 40% B at 0.15 ml/min from 1.5 to 10 min; 40% B at 0.3 ml/min from 10.5 to 14.5 min; and finally, 85% B at 0.15 ml/min from 15 to 20 min. The mass spectrometer was operated in positive ionization mode, capturing a MS1 scan with a resolution of 70,000, an automatic gain control target of 3 × 10^6^, and a scan range of 60 to 186 *m/z* and 187 to 900 *m/z*. This protocol was adapted from Mentch *et al.* (2015) (127). The data for individual metabolites were processed using MAVEN (referenced from Clasquin *et al.* (2012) (128), and Melamud *et al.* (2010) (129)), with the retention times empirically determined in-house. Peak area top values were analyzed for the determination of metabolite expression. All data are presented as mean ± standard error, unless otherwise noted.

### Histone extraction and derivatization

Cellular pellets from tissue culture were suspended in 800 μl of buffer A (10 mM Tris–HCl pH7.4, 10 mM NaCl, 3 mM MgCl_2_) chilled on ice and fortified with protease and histone deacetylase inhibitors. The resulting cell homogenate was then subjected to light pestle homogenization using a 1 ml Wheaton dounce homogenizer for 40 strokes. Following centrifugation at 800*×g* for 10 min at 4 °C, the nuclei were isolated. The nuclei were then sequentially washed in ice-cold PBS (pH 7.4), resuspended in 0.4N H_2_SO_4_, and rotated for 4 h at 4 °C. After additional centrifugation, nuclear debris and non-histone proteins were removed, and the histone proteins were precipitated using trichloroacetic acid. The precipitated histone proteins were subsequently washed, dried, and dissolved in water. Insoluble debris was removed *via* centrifugation, and the resulting histone-containing supernatant was stored at −20 °C until further analyses.

For *M. musculus* tissues, the process was similar, except the initial homogenization process involved a 1 ml Wheaton dounce homogenization followed by a filtering through 100 μM mesh. The protocol for histone isolation was otherwise consistent with the one used for tissue culture.

Before LC-MS/MS analysis, purified histone proteins were buffered, labeled with d_6_-acetic anhydride, and digested with trypsin. The peptides were then further modified with phenyl isocyanate, desalted, and dried. The final products were resuspended in sample diluent and prepared for LC-MS/MS analysis.

### UPLC MS/MS analysis

Histone peptides were injected into a Dionex Ultimate3000 nanoflow HPLC with a Waters nanoAcquity UPLC C18 column, attached to a Thermo Fisher Scientific Q-Exactive mass spectrometer at 700 nl/min. The mobile phase comprised water + 0.1% formic acid (A) and acetonitrile + 0.1% formic acid (B). Histone peptides were resolved with a two-step linear gradient of 5% to 35% B over 45 min followed by 35% to 95% B over 10 min. Data were acquired using the data-independent acquisition (DIA) mode. The MS1 scan resolution = 35,000, automatic gain control target = 1 × 10^6^, and scan range = 390 to 910 *m/z*, followed by a DIA scan with a loop count of 10. DIA settings: window size = 10 *m/z*, resolution = 17,500, automatic gain control target = 1 × 10^6^, DIA maximum fill time = auto, and normalized collision energy = 30. For each cycle, one full MS1 scan was followed by 10 MS2 scans using an isolation window size of 10 *m/z*.

### Data analysis

EpiProfile 2.0 (https://www.omicsdi.org/dataset/prode/PXD004166) was employed to process the Thermo Fisher Scientific RAW files using the AcD3 module. The previously published Histone Analysis Workflow (130) was used for normalization and statistical analyses.

### Shotgun proteomics

Around 50 mg of tissue was subjected to dounce homogenization and tip-sonication in a solution of 0.5% SDS/Tris–HCl. Protein quantity was determined using the bicinchoninic acid assay, with 50 μg selected for shotgun proteomics. Sample preparation involved denaturation and alkylation *via* the addition of 10 mM DTT and 20 mM iodoacetamide, respectively, followed by detergent removal through chloroform-methanol extraction. The protein samples, once concentrated, were redissolved and tip-sonicated in a 2 M urea/NH_4_HCO_3_ solution. Subsequent trypsin digestion occurred overnight, followed by desalting and cleaning with C18 stage tips. The final step involved drying down and resuspension in sample diluent, comprised of 5% acetonitrile, 0.1% acetic acid in HPLC-grade water.

The resulting peptides were analyzed using the same LC-MS/MS system employed for histone proteomics. The mobile phase consisted of water + 0.1% formic acid (A) and acetonitrile + 0.1% formic acid (B). Peptide resolution was achieved with a two-step linear-gradient, ranging from 2% to 35% B over 90 min and then from 35% to 95% B over 12 min. Data-dependent acquisition mode was selected for all samples.

Raw data files were processed with MaxQuant (https://maxquant.net/download_asset/perseus/latest) using label-free quantification for library samples. Protein identification and quantification were accomplished using the Perseus platform with the MaxQuant library, a 2017 version of the *M. musculus* reference genome FASTA (SwissProt canonical), and default settings.

For pathway analysis, all proteins were loaded into DAVID for functional annotation clustering, using default settings, an *M. musculus* background, and medium classification stringency.

### Western blot analysis

Twenty micrograms of total protein extracted from tissue or cell pellets was separated and transferred to nitrocellulose using the XCell SureLock Mini-Cell electrophoresis system (Invitrogen), adhering to the manufacturer's protocols. The membrane was then blocked using Intercept TBS blocking buffer (LI-COR, cat# 927-60001) and all subsequent steps were performed in Tris-buffered saline with Tween-20 + 5% bovine serum albumin. The membranes were incubated with the primary antibody overnight, then washed, and incubated for 1 h with the IRDye IgG secondary antibody. These IRDye antibodies, designed to fluoresce at two different wavelengths, facilitated the simultaneous analysis of rabbit (LI-COR, cat# P/N926-32211) and mouse (LI-COR, cat# P/N925-68070) antibodies on the same blot. The procedure involved the sequential addition of primary antibodies (phosphorylated eIF2α, cat# 3597S; eIF2α, cat# 2103S; AHCY, cat# sc-271389), followed by incubation with secondary antibodies for both species. The blots were imaged using the Odyssey infrared imaging system and analyzed with Image Studio Lite (https://www.licorbio.com/image-studio-lite).

### MTT assay

The tetrazolium salt (3-(4,5-dimethylthiazol-2-yl)-2,5-diphenyltetrazolium (MTT) solution was initially prepared at a concentration of 5 mg/ml in PBS. Posttreatment, 200 μl of the MTT solution was applied to each well of a 6-well plate. The plate was then incubated in a humidified incubator at 37 °C under 5% CO_2_ conditions for 4 h. Subsequently, the MTT solution was carefully removed, and an equal volume of acidified isopropanol (0.4N HCl) was introduced into each well. The plate was vigorously shaken at room temperature for 30 to 60 min or until the formed formazan salt was completely dissolved. The optical density of each well was measured spectrophotometrically at 570 nm.

### Crystal violet staining

Cell culture media was carefully removed, and the cells were fixed by adding 500 μl of 100% methanol, ensuring full coverage of the well. The plate was then left undisturbed at room temperature for 20 min. After the fixation step, methanol was removed, and 500 μl of staining solution, composed of 0.5% crystal violet in 25% methanol, was added to each well. This was left at room temperature for 5 min. After staining, the solution was removed, and the wells were washed twice with tap water. The plate was then left inverted to dry overnight. Qualitative colony counts were conducted on the following day using a scanner.

### Cloning, expression, and purification of human full-length AHCY

#### Cloning of human AHCY gene

Human AHCY was codon optimized to express in *Escherichia coli* and that gBlock was ordered from Intergrated DNA Technologies. (AHCY sequence:

ATGTCTGACAAACTGCCGTACAAAGTTGCTGACATCGGTCTGGCTGCTTGGGGTCGTAAAGCTCTGGACATCGCTGAAAACGAAATGCCGGGTCTGATGCGTATGCGTGAACGTTACTCTGCTTCTAAACCGCTGAAAGGTGCTCGTATCGCTGGTTGCCTGCACATGACCGTTGAAACCGCTGTTCTGATCGAAACCCTGGTTACCCTGGGTGCTGAAGTTCAGTGGTCTTCTTGCAACATCTTCTCTACCCAGGACCACGCTGCTGCTGCTATCGCTAAAGCTGGTATCCCGGTTTACGCTTGGAAAGGTGAAACCGACGAAGAATACCTGTGGTGCATCGAACAGACCCTGTACTTCAAAGACGGTCCGCTGAACATGATCCTGGACGACGGTGGTGACCTGACCAACCTGATCCACACCAAATACCCGCAGCTGCTGCCGGGTATCCGTGGTATCTCTGAAGAAACCACCACCGGTGTTCACAACCTGTACAAAATGATGGCTAACGGTATCCTGAAAGTTCCGGCTATCAACGTTAACGACTCTGTTACCAAATCTAAATTCGACAACCTGTACGGTTGCCGTGAATCTCTGATCGACGGTATCAAACGTGCTACCGACGTTATGATCGCTGGTAAAGTTGCTGTTGTTGCTGGTTACGGTGACGTTGGTAAAGGTTGCGCTCAGGCTCTGCGTGGTTTCGGTGCTCGTGTTATCATCACCGAAATCGACCCGATCAACGCTCTGCAGGCTGCTATGGAAGGTTACGAAGTTACCACCATGGACGAAGCTTGCCAGGAAGGTAACATCTTCGTTACCACCACCGGTTGCATCGACATCATCCTGGGTCGTCACTTCGAACAGATGAAAGACGACGCTATCGTTTGCAACATCGGTCACTTCGACGTTGAAATCGACGTTAAATGGCTGAACGAAAACGCTGTTGAAAAAGTTAACATCAAACCGCAGGTTGACCGTTACCGTCTGAAAAACGGTCGTCGTATCATCCTGCTGGCTGAAGGTCGTCTGGTTAACCTGGGTTGCGCTATGGGTCACCCGTCTTTCGTTATGTCTAACTCTTTCACCAACCAGGTTATGGCTCAGATCGAACTGTGGACCCACCCGGACAAATACCCGGTTGGTGTTCACTTCCTGCCGAAAAAACTGGACGAAGCTGTTGCTGAAGCTCACCTGGGTAAACTGAACGTTAAACTGACCAAACTGACCGAAAAACAGGCTCAGTACCTGGGTATGTCTTGCGACGGTCCGTTCAAACCGGACCACTACCGTTAC). The genomic sequence encoding AHCY was amplified by PCR using oligodeoxyribonucleotide primers containing restriction enzyme sites for Xho1 and Nde1. The sequences of forward and reverse primers used were 5′ CTGGTGCCGCGCGGCAGCCATATGTCTGACAAACTGCCGTAC 3′ and 3′ GTGGTGGTGGTGGTGGTGTTAGTAACGGTAGTGGTCCGGTTTG 5′, respectively.

### Construction of the recombinant expression plasmid

The PCR product of AHCY was purified by Qiagen PCR Purification Kit and ligated to the expression vector pET28a, which had previously been linearized with Xho1 and Nde1, by HiFi assembly kit. This vector encodes an N-terminal 6xHis tag, thus enabling the expressed protein to be purified by affinity chromatography. The recombinant expression vector was then transformed into the *E. coli* stellar cells. The orientation of the insert and its insertion in frame with the vector were verified by whole plasmid Sanger sequencing.

### Bacterial cell transformation and preparation of cell-free extract

Once transformed, *E. coli* BL21 cells were grown overnight in 2xYT medium containing 50 μgml^−1^ kanamycin. For induction experiments, 200 μl of the original overnight culture and grown for 2 h at 37 °C with constant shaking. Each culture was induced with various IPTG final concentrations (0.2 mM and 1 mM) and grown at various temperatures (16 °C and 37 °C) for different times (2, 4, and 16 h) with constant shaking. The growth was stopped with ice and cells were harvested by centrifugation at 4 °C. The cell pellet was lysed in the lysis buffer (50 mM Hepes, 150 mM NaCl, 10% glycerol, 20 uM leupeptin, 5 uM pepstatin-A, 0.5uM aprotinin, 50 mg/l lysozyme) and sonicated. Soluble supernatant and insoluble pellet were analyzed under denaturing conditions on a Coomassie staining blue and 10% SDS-PAGE.

For large-scale culture preparation, transformed cells were picked and then were grown overnight at 37 °C in 10 ml of 2xYT medium. Once the *A*_600_ of the cells reached 0.4 to 0.6, protein production was induced by the addition of 0.2 mM IPTG to the media, and the cells were allowed to grow overnight at 16 °C. The following morning, cells were harvested by centrifugation at 4 °C and stored at −80 °C.

### Purification of recombinant AHCY and protein analysis

The cell pellet corresponding to 1 L of culture was resuspended in 50 mM Hepes pH 7.2 and 150 mM NaCl supplemented with EDTA-free HALT Protease Inhibitor Cocktail and lysozyme and lysed by sonication. The soluble fraction of the cell lysate was isolated by high-speed centrifugation, 45 μM syringe filtered, and loaded onto a HisTrap FF chelating affinity column (1 ml, GE Healthcare) equilibrated with wash buffer (50 mM Hepes, 500 mM NaCl, 2 mM beta-mercaptoethanol, pH 7.2) and 20 mM imidazole, using an AKTA-FPLC system (GE Healthcare). The bound enzyme was eluted with the wash buffer in a gradient from 20 to 300 mM imidazole over 10 column volumes. The fractions from the gel filtration column were analyzed by SDS-PAGE, and the peak fractions were pooled and concentrated using an Amicon protein concentrator (10-kDa cutoff) until less than 10 ml for snakeskin dialysis. The concentrated sample was desalted in the dialysis buffer (20 mM Tris–HCl, 150 ml NaCl, 1 mM DTT, and 10% glycerol) overnight at 4 °C. The desalted sample was centrifuged at 21,000×*g* for 2 min at 4 °C to remove precipitation and determined the concentration using its *A*_280_ and extinction coefficient, assuming all cysteine reduced (55,350 M^−1^ cm^−1^). The protein was then divided into small aliquots, flash-frozen in liquid nitrogen, and stored at −80 °C.

### Enzymatic assay for hydrolytic activity of recombinant AHCY

The enzyme hydrolytic reaction was conducted in an assay buffer consisting of 25 mM Tris–HCl (pH 7.5), 50 μM nicotinamide adenine dinucleotide, 1 mM DTT, 1 mM EDTA, 0.01% Tween 20, and 0.01% bovine serum albumin, in a microcentrifuge tube with a final volume of 20 μl. To initiate the time-course reaction, 1 mM SAH equilibrated at 30 °C for 10 min was added to the enzyme mixture in the assay buffer. The reaction mixture was incubated at 30 °C for 120 min. For other kinetic experiments, reactions were carried out by initiating with AHCY. To prevent reaction reversal, reactions were performed in the presence of 0.2 units of ADA (Roche Applied Science) using a direct, discontinuous assay by quantifying adenosine and inosine formation from SAH. The reaction was terminated by adding 60 μl of 1% formic acid in deionized water. The microcentrifuge tubes were centrifuged at 16,000*×g* for 5 min, and the supernatant was transferred to LC-grade vials.

### LC-MS analysis

The formation of adenosine and inosine was measured using LC-MS. The LC system utilized a mobile phase (A) consisting of 97% H_2_O, 3% acetonitrile, 20 mM ammonium acetate, 15 mM ammonium hydroxide (pH 9.2), and an organic phase (B) consisting of 100% acetonitrile. Separation was carried out at a flow rate of 1 ml/min using the following linear gradient: 0 min, 85% B at 0.15 ml/min; 1.5 min, 40% B at 0.15 ml/min; 3.5 min, 40% B at 0.15 ml/min; 4 min, 40% B at 0.3 ml/min; 5 min, 40% B at 0.3 ml/min; 5.5 min, 85% B at 0.15 ml/min; and 8 min, 85% B at 0.15 ml/min.

Peak quantification of adenosine and inosine was performed using El-Maven, and absolute concentrations were calculated based on the standard calibration curves for respective metabolite.

### Inhibition analysis

Prism version 10 (https://www.graphpad.com) was used to fit the inhibition curve. The equation for noncompetitive inhibition is Y = Vmax_inh_ ∗ X/(Km + X), where Y is the enzyme velocity, X is the substrate concentration, Vmax_inh_ is the maximum enzyme velocity in the presence of the inhibitor, and Km is the Michaelis–Menten constant.

### Generation of stable cell line expressing MAT2A OE

The pCDH-Flag-MAT2A plasmid was generously provided by Dr Spencer Haws from the Denu Lab and was sequence-verified by Plasmidsaurus. To produce lentivirus, four million HEK293T cells were seeded in 15 cm dishes with Dulbecco's modified Eagle's medium (DMEM) supplemented with GlutaMAX and incubated for 24 h. For virus packaging, plasmids were prepared as follows: 2.5 μg pRSV-Rev, 2.5 μg pMD2.G, 5 μg pMDL, and 10 μg pCDH-Flag-MAT2A were diluted in room temperature OptiMEM. FuGENE 6 was mixed with the DNA-OptiMEM solution and vortexed gently. The mixture was incubated at room temperature for 20 min to allow the formation of FuGENE–DNA complexes, which were then added dropwise to the HEK293T cells. Sixteen hours posttransfection, the media were replaced with 30 ml of fresh DMEM supplemented with GlutaMAX. Viral supernatant was collected 72 h after transfection, centrifuged at 300×*g* for 5 min to remove cellular debris, and 24 ml of the clarified supernatant was transferred to a fresh 50 ml tube. The virus was concentrated following the Takara Lenti-X protocol, which included incubation for 4 h at 4 °C, followed by centrifugation at 1500×*g* for 45 min at 4 °C. The viral pellet was resuspended in 1 ml of sterile PBS, aliquoted into 100 μl single-use volumes, and flash-frozen for storage at −80 °C.

The day prior to transduction, 2 *×* 10^5^ HCT116 cells were seeded in 2 ml of DMEM in a 6-well plate. Once the cells attached and reached approximately 60% confluency, transduction was performed as follows: Lentiviral particles were thawed on ice, and Polybrene was diluted into DMEM at a final concentration of 0.004×. A transduction mixture was prepared by combining 250 μl of diluted Polybrene, 1,700 μl of DMEM, and 50 μl of lentivirus, resulting in a final Polybrene concentration of 5 μg/ml. The culture medium was removed from the wells, and the lentivirus/Polybrene mixture was added to the cells. After 48 h, the virus-containing medium was replaced with DMEM supplemented with puromycin for antibiotic selection. Selection was carried out for 72 h, after which the surviving cells were either frozen or expanded for further use.

## Data availability

All data described in this article is contained in the main text or supplementary materials. The mass spectrometry proteomics data has been deposited in MassIVE and can be accessed with username: MSV_000098325 and password: MSV_TMAO.

## Supporting information

This article contains supporting information.

## Conflict of interests

J. M. D. is a cofounder of Galilei Biosciences and a consultant for Evrys Bio. The other authors declare that they have no conflicts of interest with the contents of this article.
