# Supplementary material for: Gut microbiota–derived metabolite trimethylamine N-oxide alters the host epigenome through inhibition of S-adenosylhomocysteine hydrolase
Source: J Biol Chem. 2025 Jul 25;301(9):110521. doi: 10.1016/j.jbc.2025.110521 (PMC12390942; doi:10.1016/j.jbc.2025.110521)
Supplement: Supporting Figures [file mmc1.pdf]

Supplementary Figure 1 - Related to Figure 2

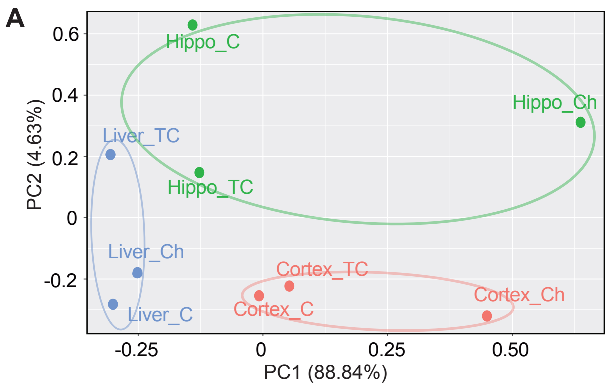

Supplementary Figure 2 - Related to Figure 3

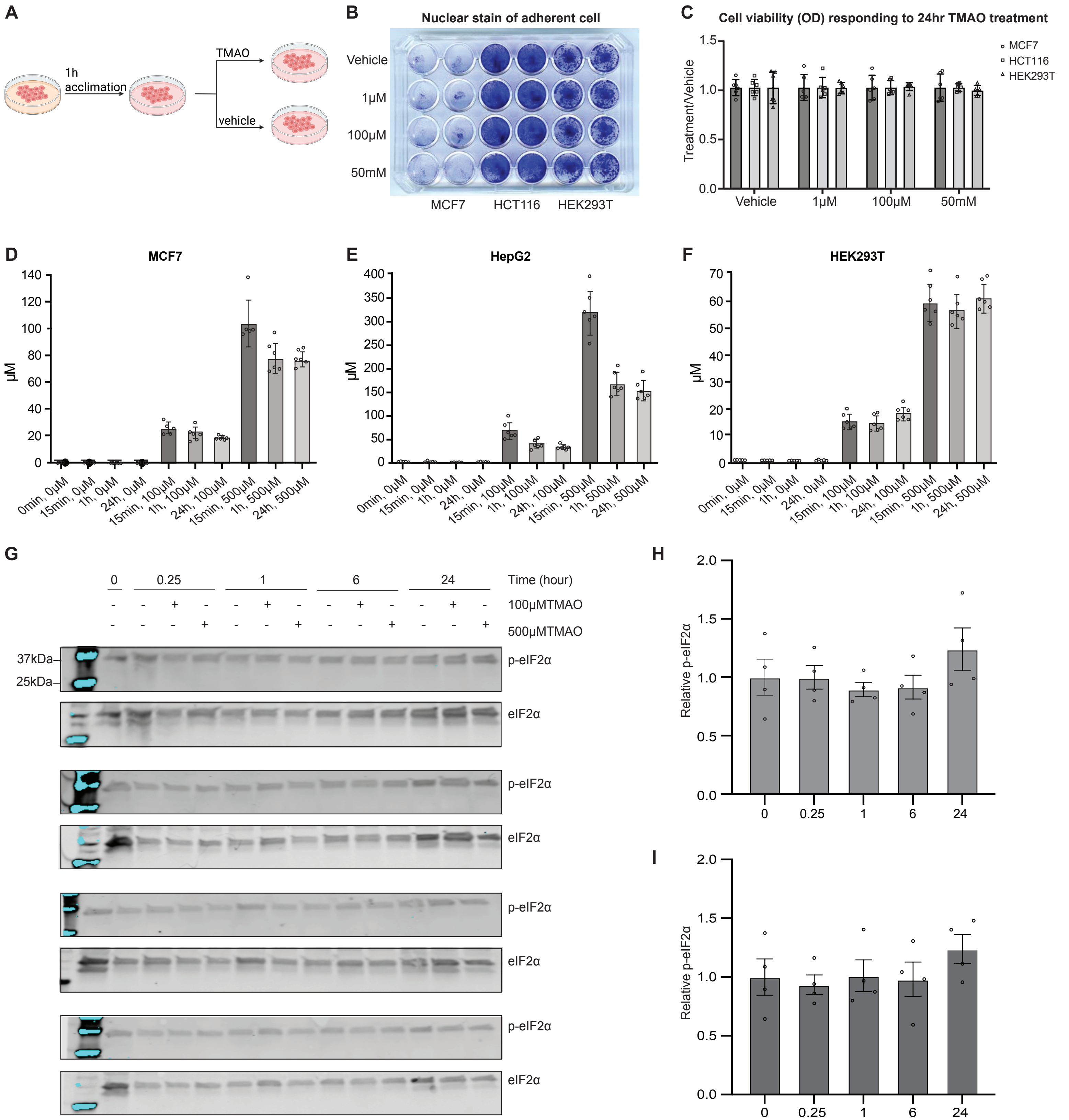

Supplementary Figure 3 - Related to Figure 4

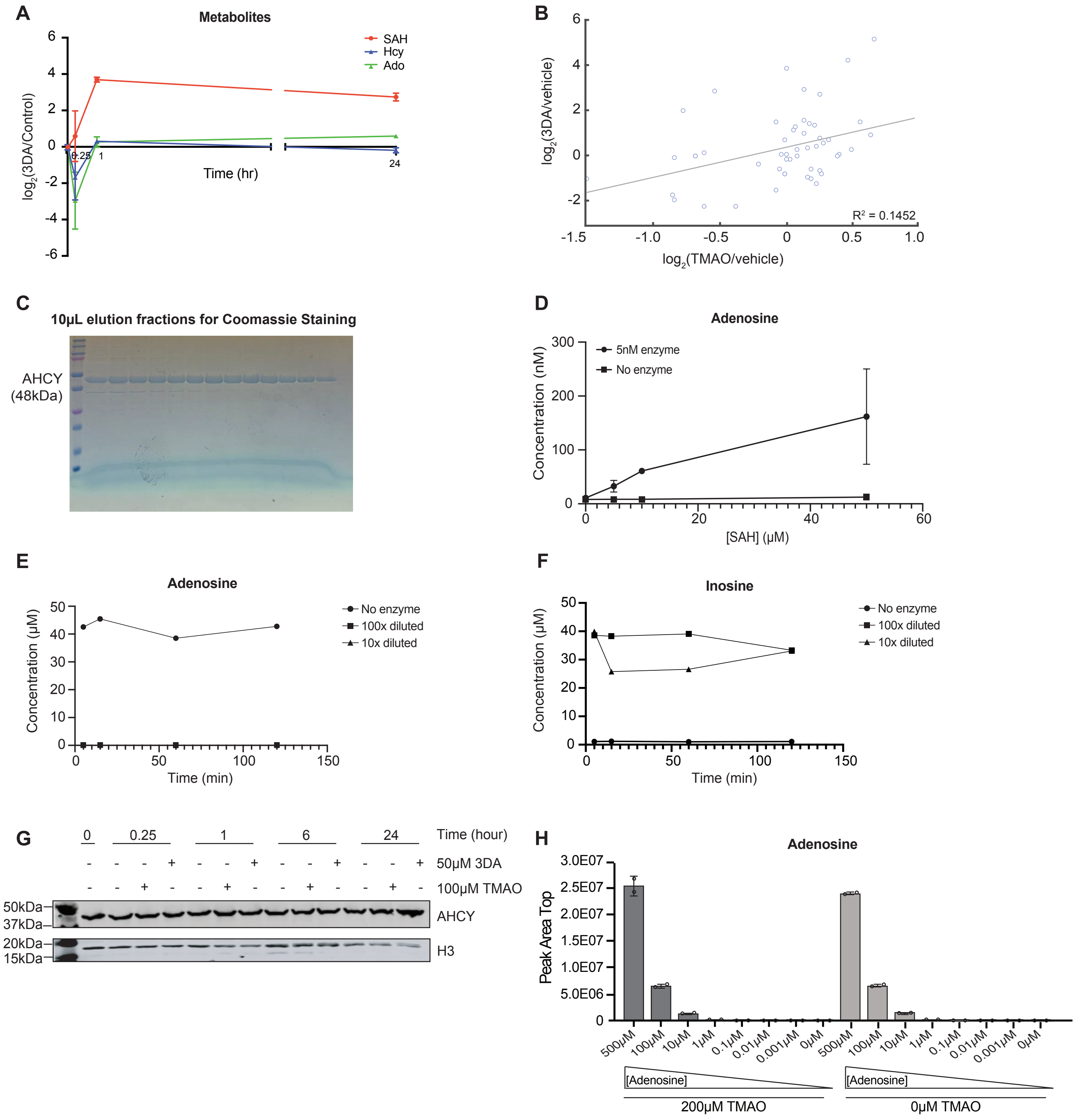

Supplementary Table - Related to Figure 4

[TMAO] (μM)	Vmax (nM/min)	k_{cat} (min^{-1})	K_M (μM)
0	13.95 ± 3.94	2.79 ± 0.79	95.57 ± 7.11
10	10.82 ± 0.81	2.16 ± 0.16	96.88 ± 2.93
50	7.59 ± 1.29	1.52 ± 0.26	52.87 ± 1.79
250	4.56 ± 2.85	0.91 ± 0.57	44.20 ± 4.46

Supplementary Figure 4 - Related to Figure 5

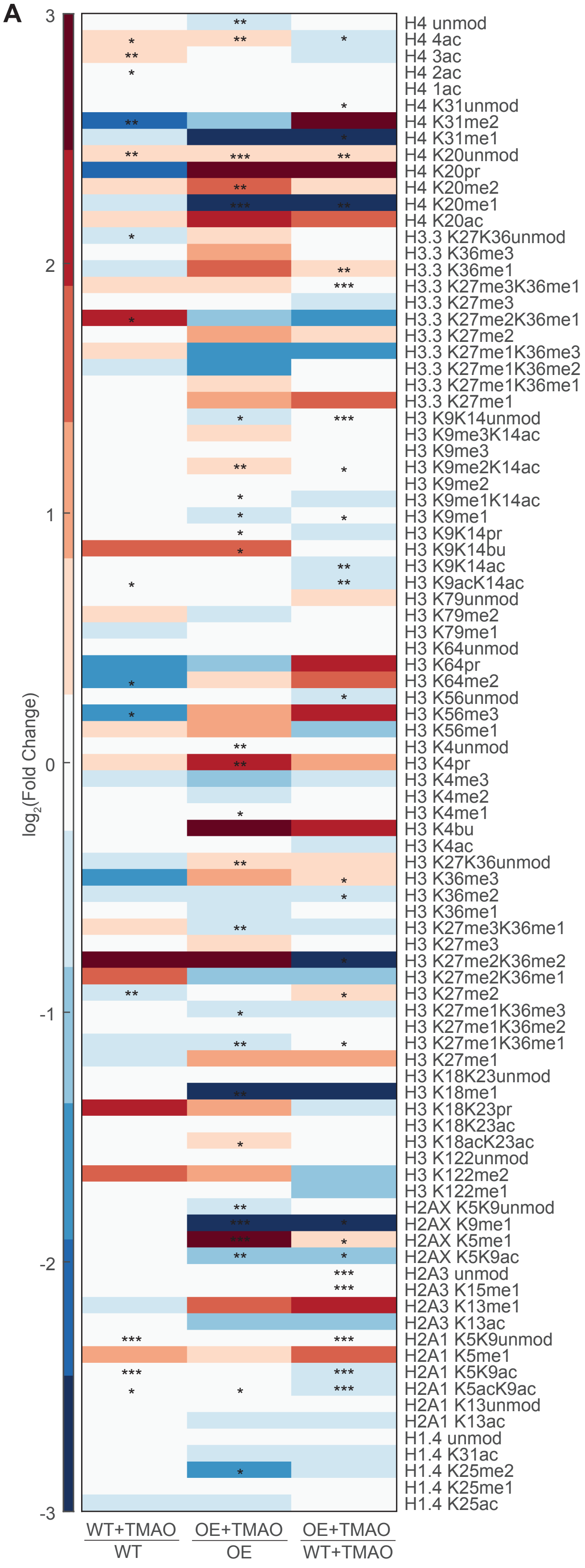

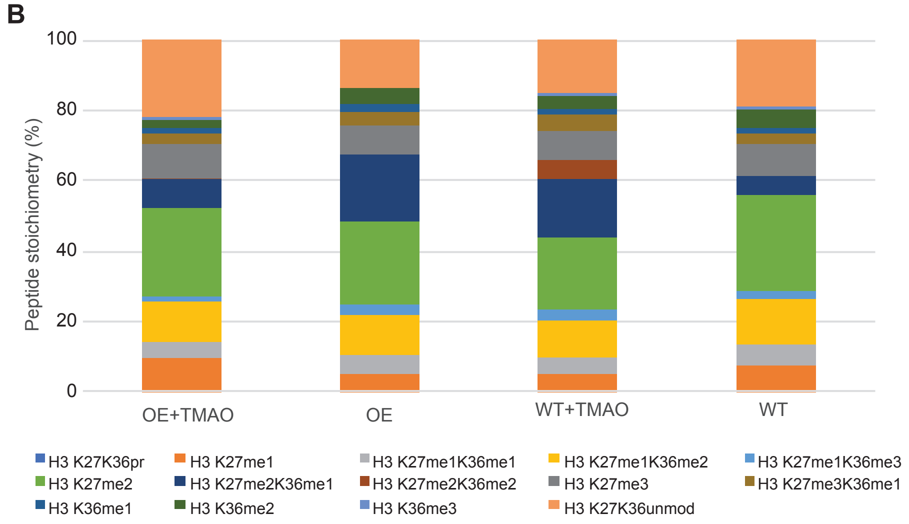

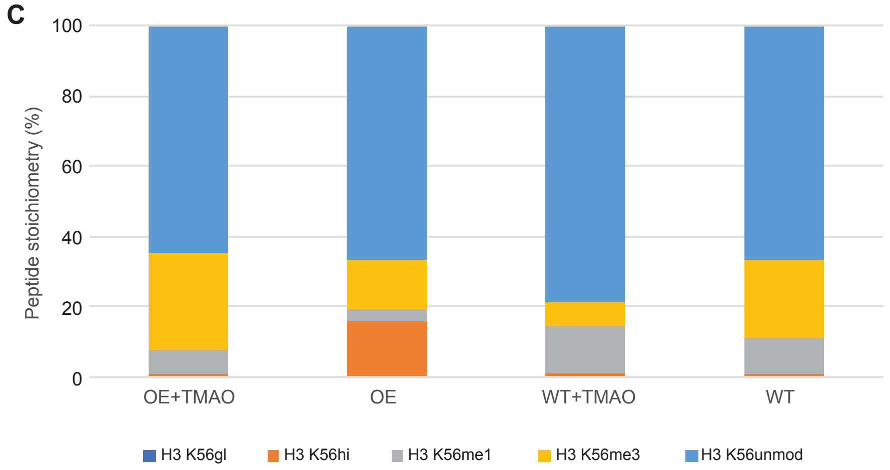

Figure Legends:

Supplementary Figure 1 – Related to Figure 2
(A) Principal component analysis (PCA) of averaged histone PTM profiles in hippocampus, cortex, and liver of mice assigned to three dietary groups: (1) standard chow (control), (2) high-choline diet (1% choline supplementation), and (3) standard chow plus 0.3% w/v TMAO in drinking water. Each point represents the dimensionally reduced, average histone PTM profile for a given tissue under each dietary condition. Ellipses indicate 95% confidence intervals, reflecting the distribution and clustering of samples. Colors correspond to tissue type.

Supplementary Figure 2 – Related to Figure 3
(A) Experimental schematic using human cell lines acclimated in treating media for 1 hour before exposure to TMAO or vehicle control. (B-C) Cell viability and proliferation, assessed in three human cell lines (MCF7, HCT116, HEK293T) over 24 hours using qualitative (crystal violet staining) and quantitative (MTT) assays, respectively. (D-F) Intracellular TMAO concentrations in MCF7, HepG2, and HEK293T cells. (G) Western blot images (H-I) Quantification of phosphorylated eIF2α and total eIF2α levels in HCT116 cells treated with 100μM or 500μM TMAO, respectively, compared to time matched control.

Supplementary Figure 3 – Related to Figure 4
(A) Time-series analysis of key methionine cycle metabolites in HCT116 cells exposed to 50μM 3DA relative to vehicle control. (B) Correlation plot of LC-MS/MS-generated log2 fold-change stoichiometric values for individual histone peptide proteoforms between 50μM 3DA treatment (30min) and 500μM TMAO treatment (15min), each compared to their time-matched vehicle controls (n=4/group). (C) Coomassie-stained SDS-PAGE gel showing 10μL of the elution fraction from AHCY purification. (D) Determination of non-enzymatic hydrolysis of adenosine at varying substrate concentrations with and without 5nM AHCY. (E-F) Quantification of adenosine (substrate) and inosine (product) in the presence of various [ADA] over time. (G) Western blots showing AHCY and total histone H3 levels following 50μM 3DA or 100μM TMAO treatment over time. (H) Quantification of adenosine after a 12-minute incubation at varying adenosine concentrations, with or without TMAO.

Supplementary Table – Related to Figure 4
AHCY Michaelis-Menten constants with titrated TMAO. Prism version 10 was used to fit the inhibition curve. The equation for noncompetitive inhibition is Y=Vmaxinh * X/(Km+X), where Y is the enzyme velocity, X is the substrate concentration, Vmaxinh is the maximum enzyme velocity in the presence of the inhibitor, and Km is the Michaelis-Menten constant. Global kinetic parameters are estimated as follows: Vmax=12.66nM/min, 95% CI [10.51, 15.74]; Km=77.24μM, 95% CI [40.24, 156.8]; and Ki=36.5μM, 95% CI [21.4, 65.8].

Supplementary Figure 4 – Related to Figure 5
(A) Heatmaps of log2 fold-change in stoichiometric histone peptide proteoforms in WT or OE cells treated with or without 500μM TMAO (n=4). Statistical significance is indicated as *p<0.05, **p<0.01, ***p<0.005 (Welch's t-test). (B-C) Peptide stoichiometry of H3K27K36 and H3K56 in OE+TMAO, OE, WT+TMAO, and WT, respectively.
